# The role of ferroptosis in diabetic cardiovascular diseases and the intervention of active ingredients of traditional Chinese medicine

**DOI:** 10.3389/fphar.2023.1286718

**Published:** 2023-10-26

**Authors:** Xiaobing Zhang, Jing Sun, Jianying Wang, Tianwei Meng, Jianfei Yang, Yabin Zhou

**Affiliations:** ^1^ Graduate School, Heilongjiang University of Chinese Medicine, Harbin, Heilongjiang, China; ^2^ Department of Cardiovascular Medicine, First Affiliated Hospital of Heilongjiang University of Chinese Medicine, Harbin, Heilongjiang, China; ^3^ Department of Endocrinology, Hanan Branch of the Second Affiliated Hospital of Heilongjiang University of Chinese Medicine, Harbin, Heilongjiang, China

**Keywords:** ferroptosis, diabetes, cardiovascular diseases, traditional Chinese medicine, active ingredients, mechanism

## Abstract

Cardiovascular diseases (CVDs), encompassing ischaemic heart disease, cardiomyopathy, and heart failure, among others, are the most prevalent complications of diabetes and the leading cause of mortality in patients with diabetes. Cell death modalities, including apoptosis, necroptosis, and pyroptosis, have been demonstrated to be involved in the pathogenesis of CVDs. As research progresses, accumulating evidence also suggests the involvement of ferroptosis, a novel form of cell death, in the pathogenesis of CVDs. Ferroptosis, characterised by iron-dependent lipid peroxidation, which culminates in membrane rupture, may present new therapeutic targets for diabetes-related cardiovascular complications. Current treatments for CVDs, such as antihypertensive, anticoagulant, lipid-lowering, and plaque-stabilising drugs, may cause severe side effects with long-term use. Traditional Chinese medicine, with its broad range of activities and minimal side effects, is widely used in China. Numerous studies have shown that active components of Chinese medicine, such as alkaloids, polyphenols, and saponins, can prevent CVDs by regulating ferroptosis. This review summarises the recent findings on the regulatory mechanisms of active components of Chinese medicine against ferroptosis in CVDs, aiming to provide new directions and a scientific basis for targeting ferroptosis for the prevention and treatment of diabetic CVDs.

## 1 Introduction

Over the past 3 decades, the number of individuals with diabetes has quadrupled, with approximately 1 in 11 adults currently affected. According to estimates by the International Diabetes Federation, the number of individuals with diabetes is projected to rise to 642 million by 2040, with over 90% of cases to be type 2 diabetes mellitus (T2DM) (Zheng et al., 2018). Diabetes is a major risk factor for cardiovascular diseases (CVDs), which in turn are the primary cause of death in individuals with diabetes (Glovaci et al., 2019). Furthermore, once CVDs occur, diabetes exacerbates the progression and impacts prognosis of CVDs (Beckman and Creager, 2016). CVDs are the most prevalent complications of diabetes; they encompass a number of conditions in the heart or blood vessels, such as ischaemic heart disease, cardiomyopathy, and heart failure (HF), and have become the most common cause of death globally (Townsend et al., 2022). Research shows that the number of people with CVDs increased from 271 million in 1990 to 523 million in 2019, i.e., nearly doubled; the number of deaths has also steadily increased, with approximately 18.6 million people having died from CVDs in 2019 (Roth et al., 2020). Therefore, there is an urgent need to identify potential pathogenic mechanisms of CVDs and find feasible treatment targets.

Cell death is a fundamental biological process, crucial in all aspects of embryonic development, growth, and aging (Bertheloot et al., 2021). Various forms of cell death, such as apoptosis, necroptosis, and pyroptosis, have been confirmed to be closely related to the pathogenesis of CVDs (Del Re et al., 2019). Increasing evidence shows that ferroptosis is also involved in the pathogenesis of CVDs (Fang et al., 2023). The concept of ferroptosis was first proposed by Dixon in 2012 (Dixon et al., 2012). Different from previously described forms of cell death, ferroptosis is a form of nonapoptotic cell death caused by iron-dependent lipid peroxidation, leading to membrane rupture (Chen et al., 2021a). This process can be inhibited by iron chelators or lipophilic antioxidants but not by inhibitors of apoptosis or necrosis (Stockwell et al., 2017; Hong et al., 2022). Cells undergoing ferroptosis show morphological characteristics such as mitochondrial shrinkage, outer membrane rupture, and a reduction or disappearance of mitochondrial cristae, while the cell nucleus is normal in size, without chromatin condensation (Mou et al., 2019; Lei et al., 2022). All these signs indicate that ferroptosis is a new form of cell death.

Therefore, studying the mechanism of ferroptosis may allow finding new targets for the treatment of CVDs. Currently, antihypertensive, anticoagulant, lipid-lowering, and plaque-stabilising drugs are commonly used in the clinical treatment of CVDs (Visseren et al., 2021). However, the side effects of long-term use of these drugs and the development of drug resistance show the urgent need to seek new treatment approaches. Traditional Chinese medicine (TCM) has a long history, and current evidence shows a definite efficacy of Chinese medicines in treating coronary atherosclerotic heart disease, HF, and other CVDs, with minimal toxic side effects (Hao et al., 2017; Meng et al., 2022). However, the mechanisms of CVDs are complex, and Chinese medicines comprise multiple components and have multiple targets. The specific role of ferroptosis in the treatment of CVDs with Chinese medicines is not yet clear. Therefore, this review discusses the mechanisms of ferroptosis and summarises the related mechanisms of active components of Chinese medicines in preventing and treating CVDs by targeting ferroptosis, in order to provide a new direction for the treatment of diabetic CVDs.

## 2 Ferroptosis and its regulatory mechanisms

**FIGURE 1:**
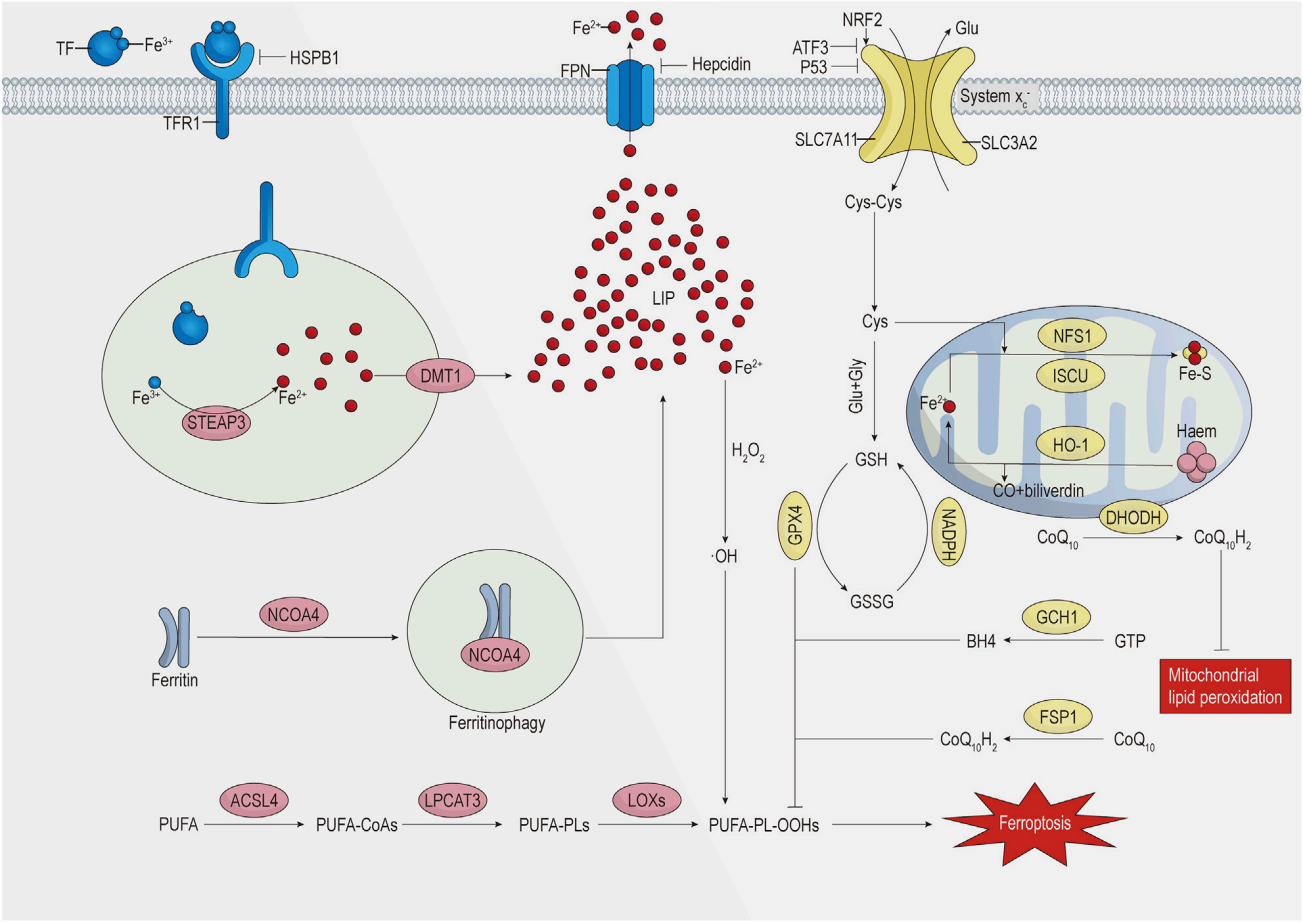
Schematic representation of the regulatory mechanism of ferroptosis.

The key to ferroptosis is the Fenton reaction caused by iron overload in cells, which leads to massive generation of hydroxyl radicals (^•^OH) and reactive oxygen species (ROS) and to the oxidation of polyunsaturated fatty acid (PUFA)-containing phospholipids (PUFA-PLs) (Lei et al., 2022). This process mainly involves metabolic pathways of iron, lipid, and amino acid metabolism (Shi et al., 2021). The specific details are shown in Figure 1.

### 2.1 Iron metabolism and ferroptosis

Iron is one of the essential trace elements in the human body. The ability of iron to provide or accept electrons in the intra- and extracellular environments makes it highly reactive and toxic (Ganz, 2013; Nemeth and Ganz, 2021). The maintenance of iron homeostasis is of great significance. Cells mainly take in iron through endocytosis. The complex formed by the binding of transferrin (TF), carrying two ferric ions (Fe3+), to TF receptor 1 (TFR1) on the cell membrane enters the cell through receptor-mediated endocytosis, which is dependent on the clathrin protein (Kawabata, 2019; Zeidan et al., 2021). Under acidic conditions of the endosome, Fe3+ separates from TF and is reduced to the ferrous ion (Fe2+) by six-transmembrane epithelial antigen of prostate 3 (STEAP3), and then Fe2+ is transported to the unstable iron pool in the cytoplasm by divalent metal transporter 1 (DMT1, also known as NRAMP2 and SLC11A2) (Vogt et al., 2021). The TF–TFR1 complex in the endosome is transported to the cell surface and reused by the cell (Kawabata, 2019). Knocking down TFR1 could inhibit cardiomyocyte ferroptosis induced by ischaemia/reperfusion (I/R) injury in rat hearts (Tang et al., 2021b). In addition, heat shock protein family B (small) member 1 (HSPB1) can inhibit the expression of TFR1 to reduce cellular iron levels. Overexpression of HSPB1 can inhibit erastin-induced ferroptosis (Sun et al., 2015).

Cytoplasmic iron can be utilised for the synthesis of haem and iron–sulphur clusters in mitochondria or stored in ferritin (Galaris et al., 2019), a complex composed of 24 ferritin heavy chain 1 (FTH1) and ferritin light chain (FTL) subunits. FTH1 possesses ferroxidase activity, which enables the conversion of Fe2+ to Fe3+ for storage (Ganz, 2013; Zhang N. et al., 2021), thereby preventing the Fenton reaction between Fe2+ and hydrogen peroxide and reducing ROS generation. Nuclear receptor coactivator 4 (NCOA4) interacts with FTH1, mediating the degradation of ferritin in autophagosomes to subsequently release its iron content into the labile iron pool, a process termed ferritinophagy (Muckenthaler et al., 2017; Ajoolabady et al., 2021). Specific knockout of the *Fth1* gene in mouse cardiomyocytes increases ROS accumulation, thereby increasing the sensitivity to ferroptosis (Fang X. et al., 2020). Conversely, knocking down NCOA4 can reduce intracellular iron levels and ROS accumulation, alleviating endothelial damage (Qin et al., 2021).

Hepcidin, a peptide hormone secreted by the liver, induces the degradation of ferroportin (FPN) (Nemeth and Ganz, 2021), the only known cellular iron export protein (Billesbølle et al., 2020). The hepcidin/FPN axis plays a crucial role in maintaining cellular iron homeostasis. Recent studies have shown that FPN is also present in cardiomyocytes. Specific knockout of *Fpn* in mouse cardiomyocytes leads to iron overload and severe cardiac damage, whereas mice with the hepcidin gene knocked out exhibit a milder phenotype and a longer survival time (Lakhal-Littleton et al., 2015; Lakhal-Littleton et al., 2016).

The iron-responsive element/iron regulatory protein (IRE/IRP) regulatory system regulates iron homeostasis at the transcriptional level. When cells are iron deficient, IRP1 or IRP2 binds to the IREs in the 3′untranslated regions (UTRs) of *TFR1* mRNA and *DMT1* mRNA, stabilising transcription and enhancing translation. Conversely, IRP1 or IRP2 binds to the IREs in the 5′UTRs of *FTH1* mRNA and *FPN1* mRNA, inhibiting translation. When cellular iron is sufficient, IRP1 assembles a cubane Fe–S cluster, preventing its binding to IREs, while IRP2 is degraded, thereby inhibiting iron accumulation (Muckenthaler et al., 2008; Anderson and Frazer, 2017). Research has confirmed that overexpression of IRP1 significantly promotes melanoma cell ferroptosis induced by erastin and RSL3 (Yao et al., 2021).

Mitochondria are crucial for the synthesis of haem and iron–sulphur clusters, thus playing a vital role in maintaining cellular iron homeostasis (Ali et al., 2022). Mitoferrin 1 (also known as SLC25A37) and mitoferrin 2 (also known as SLC25A28) are key mitochondrial iron import proteins. Knocking down mitoferrin 1 can reduce mitochondrial iron levels, decrease ROS generation, and inhibit ferroptosis (Huang et al., 2018). Haem oxygenase 1 (HO-1) degrades haem into CO, Fe^2+^, and biliverdin; HO-1 expression is regulated by nuclear factor-erythroid 2-related factor 2 (NRF2) (Zhang et al., 2020). However, whether HO-1 promotes or inhibits ferroptosis remains a matter of debate among researchers (Cai et al., 2022; Shi J. et al., 2023; Liu et al., 2023; Ni et al., 2023). Furthermore, several mitochondrial proteins involved in the synthesis of iron–sulphur clusters, such as cysteine desulfurase (NFS1), iron–sulphur cluster assembly enzyme (ISCU), CDGSH iron sulphur domain 1 (CISD1), and CDGSH iron sulphur domain 2 (CISD2), can inhibit ferroptosis by reducing mitochondrial Fe^2+^ levels and decreasing ROS production (Chen et al., 2021a).

### 2.2 Lipid metabolism and ferroptosis

PLs are crucial components of the cell membrane, and the peroxidation of PUFA-PLs is a prerequisite for ferroptosis (Wu et al., 2021). Owing to the weak C–H bond at the *bis*-allylic position of PUFAs, they are susceptible to peroxidation (Stockwell, 2022). Increasing the cellular content of PUFAs increases the sensitivity to ferroptosis (Liang D. et al., 2022). However, free PUFAs cannot directly cause ferroptosis; they need to be integrated into PLs of the plasma membrane to drive ferroptosis (Li J. et al., 2020; Stockwell, 2022). Monounsaturated fatty acids (MUFAs) can compete with PUFAs for the incorporation into PLs. Since MUFAs lack a *bis*-allylic structure, they are less prone to peroxidation (Naowarojna et al., 2023). Increasing the MUFA content can reduce the sensitivity to ferroptosis (Das, 2019). Acyl-coenzyme A (CoA) synthetase long-chain family member 3 (ACSL3) mediates the activation of MUFAs and inserts them into PLs (Pope and Dixon, 2023). The absence of ACSL3 can increase the sensitivity of melanoma cells to ferroptosis (Ubellacker et al., 2020).

Unlike ACSL3, acyl-CoA synthetase long-chain family member 4 (ACSL4) is capable of catalysing the binding of PUFAs (particularly arachidonic and adrenic acids) to CoA to generate PUFA-CoAs. Subsequently, lysophosphatidylcholine acyltransferase 3 (LPCAT3) incorporates them into PLs, forming PUFA-PLs (primarily arachidonic acid-phosphatidylethanolamines and adrenic acid-phosphatidylethanolamines) (Lin Z. et al., 2021; Lei et al., 2022). ACSL4 and LPCAT3 are key drivers in the production of ferroptotic lipids (Jiang et al., 2021; Stockwell, 2022), while the inactivation of ACSL4 or LPCAT3 reduces or inhibits ferroptosis. For instance, the knockout of *Acsl4* significantly reduces ferroptosis in mice with acute kidney injury (Wang Y. et al., 2022), while inhibition of LPCAT3 protects human cells from ferroptosis (Reed et al., 2022).

PUFA-PLs form hydroperoxides (PUFA-PL-OOHs) through nonenzymatic or enzymatic oxidation reactions (Jiang et al., 2021; Shi et al., 2021). Nonenzymatic oxidation reactions are primarily triggered by the Fenton reaction product ^•^OH, and unlike strictly controlled enzymatic reactions, they are poorly controlled and prone to chain reactions (Shi et al., 2021). Lipoxygenases (LOXs) are a class of non-haem iron oxidoreductases that mediate enzymatic reactions in lipid oxidation (Chen et al., 2021b). Knocking out LOXs can inhibit erastin-induced ferroptosis (Yang et al., 2016). As the peroxidation reaction continues and a large number of secondary products are generated, the integrity of the cell membrane is compromised, triggering ferroptosis.

### 2.3 Amino acid metabolism and ferroptosis

The glutathione (GSH) peroxidase 4 (GPX4)-mediated GSH metabolic pathway is a crucial defence mechanism against ferroptosis (Seibt et al., 2019). GPX4 can reduce PUFA-PL-OOHs to nontoxic PUFA-PL alcohols (PUFA-PL-OHs), protecting the cell membrane from oxidative damage, while reduced GSH is converted into oxidised GSH (GSSG) (Imai et al., 2017). GPX4 is a selenoprotein, and its expression is regulated by the selenium content (Forcina and Dixon, 2019). Overexpression of GPX4 can inhibit RSL3-induced ferroptosis, whereas inhibition of GPX4 increases cell sensitivity to ferroptosis (Yang et al., 2014). However, some tumour cells can still resist ferroptosis after inhibition of GPX4, which indicates the existence of GPX4-independent defence pathways against ferroptosis (Bersuker et al., 2019).

GSH, a key substrate of GPX4, is a tripeptide antioxidant composed of cysteine, glutamic acid, and glycine. Reduced nicotinamide adenine dinucleotide phosphate (NADPH) can maintain GSH levels (Lu, 2009). Depletion of GSH inactivates GPX4, triggering ferroptosis (Sun et al., 2018). Cysteine is considered the rate-limiting precursor for GSH synthesis (Shi Y. et al., 2023). Although cysteine can be synthesised through the transsulphuration pathway, most cells primarily obtain cysteine through the cystine–glutamate antiporter (system x_c_^–^) (Fang et al., 2023). System x_c_^–^belongs to the heterodimeric amino acid transporter (HAT) family and is composed of the light chain subunit SLC7A11 (also known as xCT) and the heavy chain subunit SLC3A2 (also known as 4F2HC) (Liu M.-R. et al., 2021). SLC7A11 mainly mediates the transport function of the complex, while SLC3A2 primarily stabilises the complex structure and ensures proper membrane localisation (Liu M.-R. et al., 2021; Fang et al., 2023). Extracellular cystine and intracellular glutamate are exchanged at a 1:1 ratio through system x_c_^–^, with cystine being converted into cysteine in the cell to participate in GSH synthesis (Lewerenz et al., 2013). SLC7A11 has multiple regulatory factors. Activating transcription factor 3 (ATF3) can inhibit SLC7A11 activity, deplete GSH, and thus promote erastin-induced ferroptosis (Wang et al., 2020). p53, a tumour suppressor protein, can also inhibit SLC7A11 expression, increasing cell sensitivity to ferroptosis (Jiang et al., 2015). NRF2 can promote SLC7A11 expression, and knocking down NRF2 promotes the occurrence of ferroptosis (Dong et al., 2020). Additionally, as the extracellular concentration of glutamate increases, it can inhibit system x_c_^–^, thereby inducing ferroptosis, which indicates that glutamate is also a regulatory factor in ferroptosis (Fan et al., 2023).

### 2.4 Other defensive mechanisms against ferroptosis

As mentioned above, there are GPX4-independent defence mechanisms against ferroptosis. Ferroptosis suppressor protein 1 (FSP1) can convert CoQ_10_ (also known as ubiquinone) into CoQ_10_H_2_ (also known as ubiquinol). As a lipophilic antioxidant, CoQ_10_H_2_ can prevent lipid peroxidation and inhibit ferroptosis (Li et al., 2023). Similarly, dihydroorotate dehydrogenase (DHODH) converts CoQ10 on the inner mitochondrial membrane into CoQ_10_H_2_, thus inhibiting mitochondrial ferroptosis (Lei et al., 2022; Stockwell, 2022). GTP cyclohydrolase 1 (GCH1) mediates the generation of tetrahydrobiopterin (BH4). BH4 inhibits ferroptosis by capturing lipid free radicals. Additionally, GCH1 can increase the abundance of CoQ_10_H_2_ and consume PUFA-PLs to prevent ferroptosis (Jiang et al., 2021; Stockwell, 2022). These pathways provide potential targets for intervening in ferroptosis.

## 3 Ferroptosis and diabetes

Increasing evidence suggests that ferroptosis plays a significant role in the development and progression of T2DM and its complications (Deng et al., 2023). Maintaining iron homeostasis is crucial for the endocrine system, as iron metabolism is involved in various glucose pathways, including insulin secretion, hepatic metabolism, and lipid metabolism (Backe et al., 2016; Zhang Z. et al., 2021; Gao et al., 2022). Iron overload is a known risk factor for T2DM (Simcox and McClain, 2013), as excessive Fe^2+^ triggers the Fenton reaction, resulting in the production of large amounts of ROS. Owing to the weak antioxidant capacity of pancreatic β cells and low expression and activity of superoxide dismutase (SOD) and GPX4, these cells are susceptible to oxidative stress-induced damage (Wang and Wang, 2017). Ferroptosis of pancreatic β cells leads to reduced insulin synthesis and secretion, ultimately triggering diabetes (Xie et al., 2023). Abnormalities in Fe–S cluster regulation in mitochondria also contribute to ferroptosis of pancreatic β cells, leading to the development of diabetes (Miao et al., 2023). Additionally, chronic arsenic exposure has been identified as a high-risk factor for T2DM (Yang and Yang, 2022), as it causes mitochondrial damage in pancreatic β cells, resulting in excessive mitochondrial ROS production, which triggers ferroptosis and impairs insulin secretion (Xie et al., 2023). The existence of ferroptosis has been confirmed in both *in vivo* and *in vitro* models of NaAsO_2_-induced pancreatic β-cell injury (Wei et al., 2020).

## 4 Ferroptosis and CVDs

**FIGURE 2:**
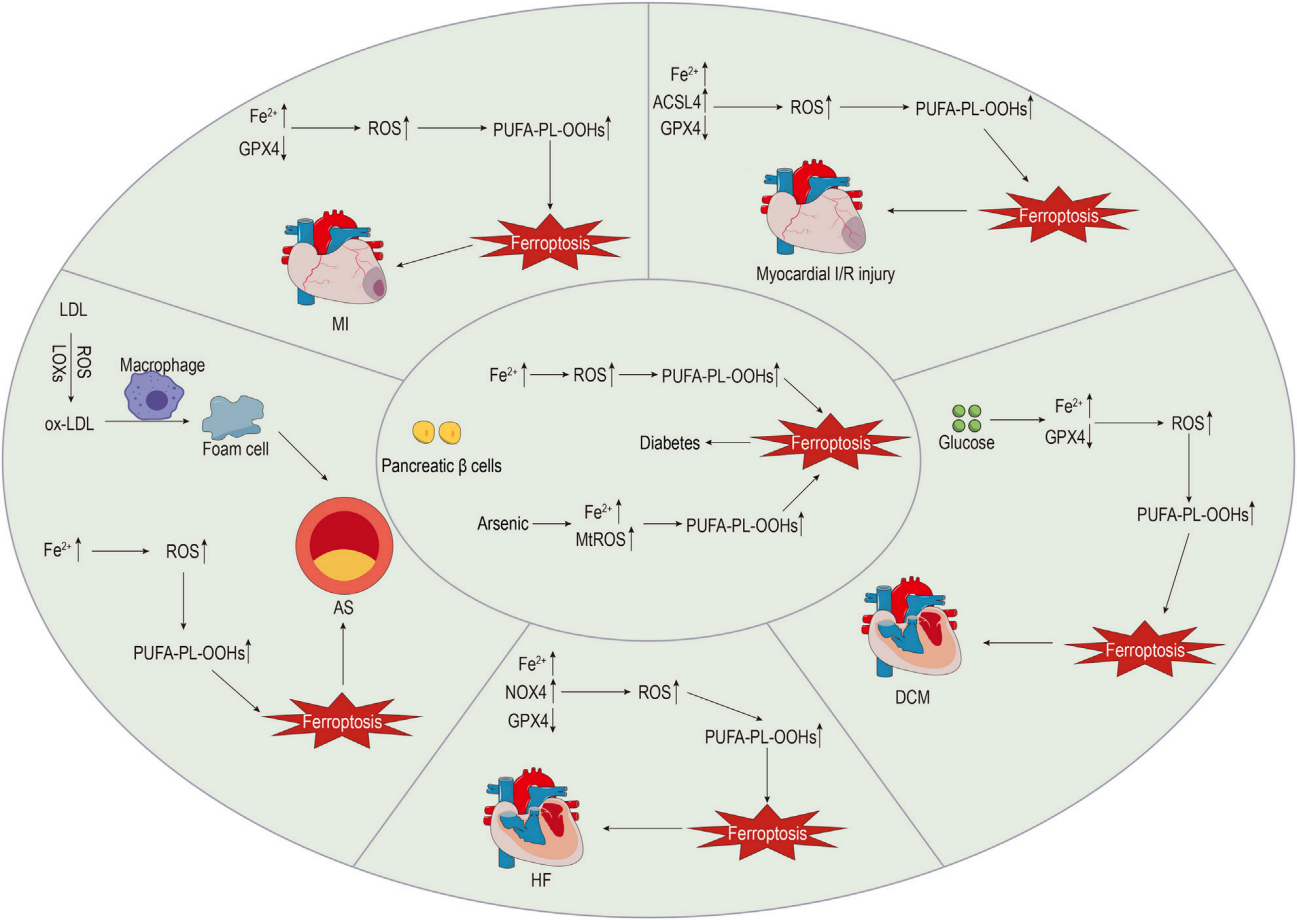
The role of ferroptosis in diabetes and CVDs.

The intricate mechanisms of CVDs make them the most prevalent cause of death. Evidence suggests that ferroptosis plays a role in the pathophysiology of CVDs, including atherosclerosis (AS), myocardial infarction (MI), myocardial I/R injury, cardiomyopathy, and HF (Wu et al., 2021). The specific details are shown in Figure 2.

Investigating the role of ferroptosis in CVDs may reveal novel therapeutic targets.

### 4.1 Ferroptosis and AS

AS is the most common macrovascular complication of diabetes, and it serves as the pathological basis for various CVDs, significantly impacting the quality of life of affected individuals (Katakami, 2018). Dysfunction of vascular endothelial and smooth muscle cells, which is a key characteristic of AS, disrupts vascular homeostasis, with long-term hyperglycaemia and insulin resistance exacerbating this process through oxidative stress and inflammatory reactions (Jyotsna et al., 2023). The deposition of lipids or fibrous material in the arterial intima gradually forms atherosclerotic plaques. The rupture of unstable plaques leads to thrombosis, ultimately resulting in vascular stenosis or occlusion (Libby et al., 2019). The pathological features of AS mainly include lipid metabolism disorders, oxidative stress, endothelial cell injury, and inflammation (Wang et al., 2021). As early as in 1994, studies indicated that iron overload exacerbated endothelial injury, thereby causing AS (Jacob, 1994). Chronic iron overload increases ROS levels in the aorta and induces oxidative stress, which leads to endothelial cell damage and promotes AS progression (Marques et al., 2019). LOXs participate in lipid peroxidation, and upregulation of 12/15-LOX expression promotes the deposition of low-density lipoprotein (LDL) beneath the vascular endothelium, where it is oxidised (Li et al., 2018). Macrophages then engulf oxidised LDL, forming foam cells and further promoting AS development (Wang et al., 2021; Ma et al., 2022). Macrophages also engulf senescent red blood cells, generating haem, which is degraded into Fe^2+^ under the action of HO-1. Iron overload and ferroptosis in macrophages accelerate AS progression (Ma et al., 2022). Research by Xu et al. (Xu et al., 2023) demonstrated that inhibiting macrophage ferroptosis through the NRF2 pathway significantly delayed the development of AS. A study by Bai et al. (Bai et al., 2020) showed that the inhibitor of ferroptosis ferrostatin-1 could alleviate AS injury in *ApoE* ^*−/−*^ mice that were fed a high-fat diet. Moreover, ferrostatin-1 could reduce iron accumulation in *ApoE* ^*−/−*^ mice, increase the expression of SLC7A11 and GPX4, and enhance the vitality of mouse arterial endothelial cells. This suggests that the use of ferroptosis inhibitors may be a new direction for AS treatment. Moreover, recent studies have discovered that ferrostatin-1 inhibits ferroptosis in vascular smooth muscle cells of high-fat diet-fed mice through a pathway independent of p53/SLC7A11/GPX4, thereby improving AS lesions (You et al., 2023). Further research has indicated that NRF2/FSP1 may be a key antioxidant target for suppressing ferroptosis in vascular smooth muscle cells, thereby providing new strategies for AS treatment (You et al., 2023). Additionally, high levels of uric acid have been shown to promote AS by inducing ferroptosis through the inhibition of the NRF2/SLC7A11/GPX4 signalling pathway (Yu et al., 2022).

### 4.2 Ferroptosis and MI

Chronic hyperglycaemia and insulin resistance negatively impact lipid metabolism, accelerating the progression of AS and increasing plaque instability (Chen et al., 2022). Following plaque rupture, thrombosis occludes the coronary artery, leading to sustained myocardial ischaemia and local myocardial necrosis (Wang and Kang, 2021). MI remains a common cause of HF, with a relatively high mortality rate, particularly in patients with concomitant diabetes (Miki et al., 2012). In MI mouse models, FTH1 levels are significantly reduced, and punctate iron deposition appears in the infarcted area (Omiya et al., 2009), suggesting a possible link between MI and ferroptosis. Using proteomic analysis, Park et al. (Park et al., 2019) found that in MI mouse models, the levels of GPX4 were significantly decreased. Specific knockout or inhibition of GPX4 leads to lipid peroxidation, promoting H9c2 cell ferroptosis, which elucidates the potential mechanism of ferroptosis in the occurrence and development of MI. A deeper understanding of the disease mechanisms can aid in discovering new treatments. By exploring the protective mechanism of mesenchymal stem cell (MSC)-derived exosomes in acute MI mouse models, Song et al. (Song et al., 2021) found that DMT1 expression was upregulated, Fe^2+^ levels increased, and GSH levels and GPX4 activity both decreased. Further research found that MSC-derived exosomes inhibited ferroptosis by targeting DMT1 expression, which reduced myocardial injury, thus demonstrating the tremendous potential of MSC-derived exosomes for MI treatment.

### 4.3 Ferroptosis and myocardial I/R injury

Prompt restoration of the blood supply to the infarcted area is the preferred treatment for MI that significantly reduces mortality. Meanwhile, prolonged myocardial ischaemia can lead to more severe myocardial injury after reperfusion, a process known as myocardial I/R injury (He et al., 2022). Both preclinical and clinical data indicate that diabetes increases susceptibility to myocardial I/R injury, attenuating protective effects of the heart and influencing patient prognosis (Russo et al., 2017), while exacerbating myocardial I/R injury through oxidative stress and other mechanisms (Zhao et al., 2017). Currently, there are no effective clinical methods to avoid myocardial I/R injury, but recent research on the mechanisms of ferroptosis and myocardial I/R injury may provide new strategies for treating myocardial I/R injury (Zhao K. et al., 2023). Tang et al. (Tang et al., 2021a) subjected rat hearts to ischaemia and reperfusion for different durations and observed that ACSL4, iron, and malondialdehyde (MDA) levels gradually increased with the reperfusion time, while GPX4 levels decreased, suggesting that myocardial cell ferroptosis mainly occurs during the reperfusion stage. This discovery could provide a basis for precise treatment of myocardial I/R injury. Cai et al. (Cai et al., 2023) used a mouse model with ligation of the left anterior descending coronary artery and similarly found that myocardial cells underwent ferroptosis during prolonged reperfusion. Researchers also found that ALOX15 expression was specifically increased in a damaged myocardium, while inhibiting ALOX15 expression could reduce myocardial cell ferroptosis, protecting the damaged myocardium. GPX4 is a key endogenous inhibitor of ferroptosis, and Sun et al. (Sun et al., 2021) found using their established rat model of myocardial I/R injury that during I/R, intracellular Fe^2+^ levels increased, GPX4 and FTH1 expression decreased, and downregulating GPX4 promoted myocardial cell ferroptosis, exacerbating myocardial injury. Additionally, research has found that myocardial I/R injury is related to endoplasmic reticulum stress (ERS), and erastin can exacerbate ERS by inducing ferroptosis. Conversely, inhibiting ERS can alleviate ferroptosis and myocardial injury (Li W. et al., 2020; Miyamoto et al., 2022). In summary, ferroptosis plays a crucial role in the pathogenesis of myocardial I/R injury and may provide new targets for treating myocardial I/R injury.

### 4.4 Ferroptosis and cardiomyopathy

Diabetic cardiomyopathy (DCM) is a severe complication of diabetes, unrelated to hypertension and coronary artery disease (Guo et al., 2022). Long-term hyperglycaemia and hyperinsulinaemia, induced by diabetes, impair capillaries, leading to myocardial fibrosis and hypertrophy. Oxidative stress and inflammatory reactions associated with diabetes also contribute to these processes (Nakamura et al., 2022). Additionally, diabetes disrupts lipid metabolism, causing excessive uptake of fatty acids by the heart. Once the storage and oxidation capacities are exceeded, this excess becomes lipotoxic, resulting in myocardial hypertrophy and dysfunction (Nakamura and Sadoshima, 2020). Research shows that ferroptosis participates in the pathophysiological process of DCM, which is mainly manifested as high ROS production and a decreased antioxidant capacity (Zhao Y. et al., 2023). Sun et al. (Sun et al., 2023) found that exogenous spermine could alleviate DCM by reducing ROS production and inhibiting ferroptosis. Wang et al. (Wang X. et al., 2022) found that sulphoraphane could activate the NRF2 signalling pathway, increase the levels of ferritin and SLC7A11 to inhibit ferroptosis, and protect cardiomyocytes. The above research indicates that the inhibition of ferroptosis is promising for the prevention and treatment of DCM.

### 4.5 Ferroptosis and HF

HF is a severe cardiac disease due to myocardial injury, wherein the cardiac output cannot meet the needs of the body, and HF is the final stage of various CVDs (Xie et al., 2022). There is a close relationship between HF and diabetes, which serves as an independent risk factor for HF. HF can develop not only from ischaemic heart disease associated with diabetes but also from DCM based on metabolic disorders, such as glucotoxicity and lipotoxicity (Nakamura et al., 2022). Research has shown that both iron deficiency and iron overload can lead to HF, and myocardial cells are highly susceptible to iron overload (Li et al., 2021). Ferroptosis of myocardial cells results in severe cardiac dysfunction, as the loss of terminally differentiated myocardial cells is irreversible. Early inhibition of ferroptosis of myocardial cells can help maintain cardiac function and delay the progression of HF (Zhang et al., 2019; Yang X. et al., 2022). In a rat model of HF induced by aortic stenosis, Liu et al. (Liu et al., 2018) found a significant increase in NADPH oxidase 4 (NOX4) levels in myocardial cells, along with decreased levels of GPX4 and FTH1. However, puerarin, an antioxidant, could reverse these phenomena, providing a promising therapeutic approach for HF. In a rat model of HF induced by aortic constriction, Chen et al. (Chen et al., 2019) found that knocking down Toll-like receptor 4 (TLR4) or NOX4 could inhibit ferroptosis and delay rat HF, suggesting potential therapeutic targets. Resveratrol is a polyphenolic substance that can inhibit the p53 pathway, reduce the degradation of SLC7A11, and increase GSH and GPX4 levels to reduce ferroptosis and improve heart function (Zhang et al., 2023).

## 5 Clinical applications of ferroptosis

Despite significant progress in the basic research of ferroptosis, the path to clinical applications has not been straightforward (von Samson-Himmelstjerna et al., 2022). Currently, the clinical applications of ferroptosis for disease diagnosis and treatment are still in their infancy.

The lack of specific biomarkers for ferroptosis has been a major limiting factor in the development of this field (Fang et al., 2023). Currently, the biomarkers used in clinical practice, such as serum iron, serum ferritin, TF, and soluble TF receptor, are nonspecific (Leng et al., 2021). Among them, serum iron is used most commonly, and monitoring its levels serves as an important indicator for assessing ferroptosis. A retrospective study has shown that elevated serum levels of iron are associated with increased severity of AS (Ozdemir, 2020). A cohort study has demonstrated a significant correlation between elevated serum ferritin levels and an increased risk of T2DM (Díaz-López et al., 2020). MDA, a lipid peroxidation product, can also be used to predict ferroptosis (Wang K. et al., 2022). Research has shown that serum MDA levels are the strongest predictor of CVD in patients on dialysis, and there is a positive correlation between serum MDA levels and the incidence of CVDs (Boaz et al., 1999). GPX4 and ACSL4 are proteins that are relatively stable in serum and have the advantages of easy measurement and sensitivity, making them recognised biomarkers for ferroptosis (Wang K. et al., 2022).

Regulators of ferroptosis include inducers and inhibitors. Based on different mechanisms of action, ferroptosis inducers can be roughly divided into iron metabolism inducers, system x_c_^–^inhibitors, and GPX4 inhibitors, which are mostly used in the treatment of tumours and neurological diseases (Xia et al., 2021). Ferroptosis inhibitors have the potential to treat CVDs and mainly include iron chelators and lipophilic antioxidants. Deferoxamine and deferasirox are iron chelators with a strong affinity for iron that are commonly used in clinical practice (Chen et al., 2023). Studies have shown that deferoxamine can increase GPX4 expression, alleviate ferroptosis, and reduce the MI area (Tu et al., 2021). In addition, dexrazoxane is the only iron chelator approved by the US Food and Drug Administration for preventing doxorubicin-induced cardiotoxicity in patients with cancer (Fang et al., 2023) by exerting a cardioprotective effect via inhibition of ferroptosis (Fang et al., 2019). Ferrostatin-1 and liproxstatin-1 are both lipophilic antioxidants, and ferrostatin-1 is the first-generation ferroptosis inhibitor (Dixon et al., 2012) that reduces myocardial I/R injury by inhibiting lipid peroxidation and improving cardiac function in diabetic mice (Li et al., 2020). Liproxstatin-1 has a similar mechanism of action and can alleviate myocardial I/R injury in mice by reducing mitochondrial ROS production and maintaining GPX4 activity (Feng et al., 2019).

## 6 Intervention with active ingredients of TCMs in ferroptosis to treat CVDs

**FIGURE 3:**
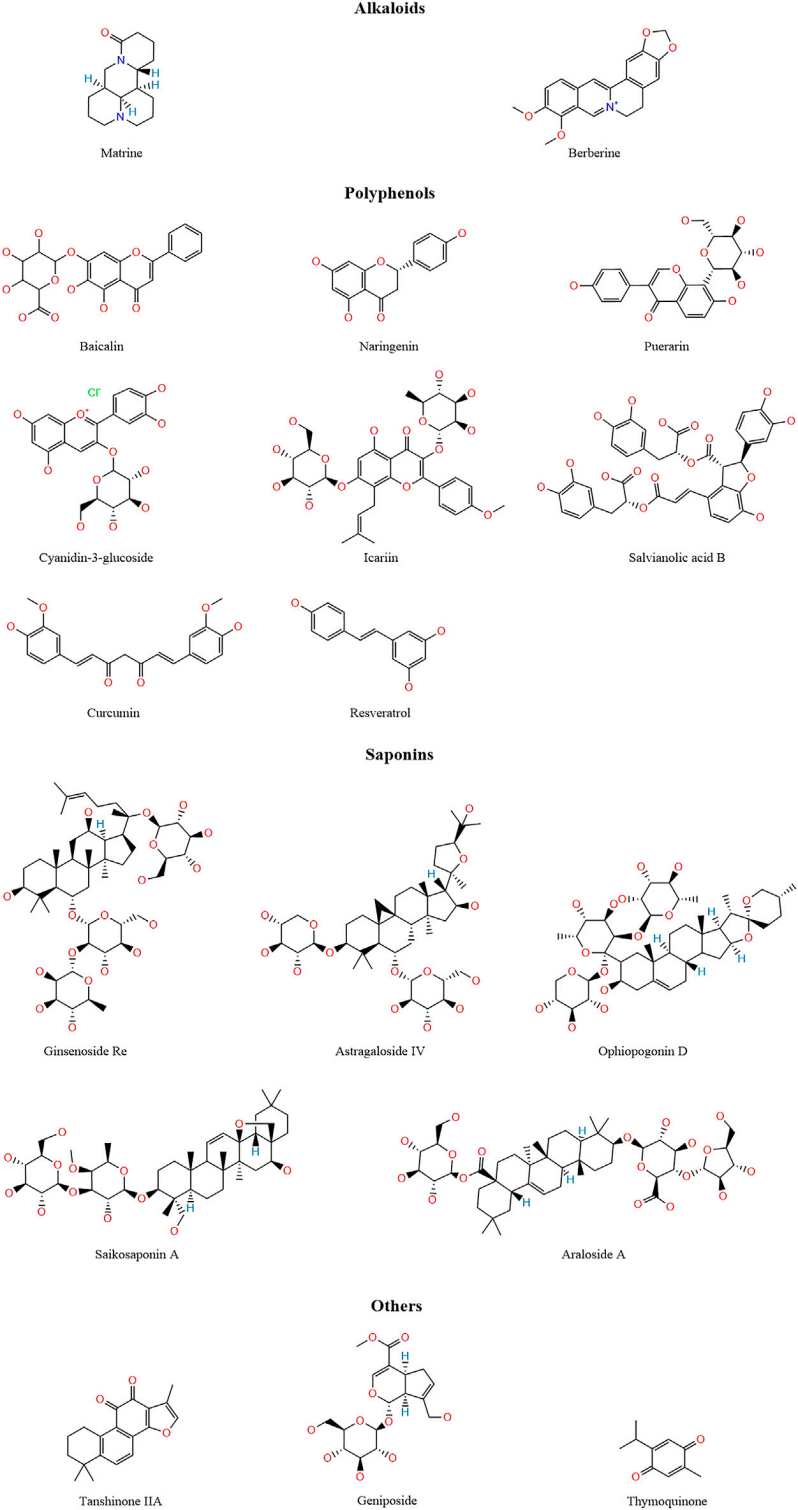
The structure of active ingredients of traditional Chinese medicine.

TCM has a wealth of clinical experience in treating CVDs, and its formulations have the advantages of low toxicity and low side effects, a wide application range, and low prices. TCMs are natural compound libraries, and many active ingredients of TCMs have been confirmed to be useful for the treatment of CVDs, with their mechanisms being related to ferroptosis. Therefore, we summarised the mechanisms of various active ingredients of TCMs in the treatment of CVDs in Table 1. Figure 3 shows the chemical structures of natural medicines.

**TABLE 1:** Mechanisms of active ingredients of traditional Chinese medicine regulating ferroptosis.

Active ingredients		Mechanism and effect	Models		Ref
			*In Vivo*	*In Vitro*	
Alkaloids	Matrine	Active the PI3K/AKT signalling pathway, upregulate the expression of GPX4, downregulate the expression of ACSL4	C57BL/6 mice	–	Xiao et al. (2023)
	Berberine	Upregulate the expression of NRF2, FTH1 and GPX4, downregulate the expression of TFR1 and p53	C57BL/6j mice	H9c2 cells	Song et al. (2023)
Polyphenols	Baicalin	Downregulate the expression of TFR1, NCOA4 and ACSL4, reduce ferritinophagy	SD rats	H9c2 cells	Fan et al. (2021)
		Upregulate the expression of GPX4	*ApoE* ^*−/−*^ mice	–	Wu et al. (2018)
	Naringenin	Upregulate the expression of NRF2, SLC7A11, GPX4, FTH1 and FPN	SD rats	H9c2 cells	Xu et al. (2021)
	Puerarin	Upregulate the expression of FTH1 and GPX4, downregulate the expression of NOX4, reduce lipid peroxidation	SD rats	H9c2 cells	Liu et al. (2018)
		Active the AMPK signalling pathway, upregulate the expression of GPX4 and ferritin, downregulate the expression of ACSL4 and TFR1	SD rats	–	Zhou et al. (2022)
		Upregulate the levels of GSH and GPX4, downregulate the levels of ROS and MDA	C57BL/6 mice	H9c2 cells	Ding et al. (2023)
	Cyanidin-3-glucoside	Upregulate the expression of FTH1 and GPX4, downregulate the expression of NCOA4 and TFR1, reduce ferritinophagy	SD rats	H9c2 cells	Shan et al. (2021)
	Icariin	Active the NRF2/HO-1 signalling pathway, upregulate the levels of GPX4, downregulate the levels of Fe^2+^ and ACSL4	–	H9c2 cells	Liu et al. (2021b)
		Active the SIRT1/NRF2/HO-1 signalling pathway, upregulate the expression of GPX4 and SLC7A11, downregulate the expression of ACSL4 and p53	C57BL/6 mice	HL-1 atrial myocytes	Yu et al. (2023)
		Upregulate the expression of GPX4 and FTH1, downregulate the expression of TFR1	*ApoE* ^*−/−*^ mice	HUVECs	Wang et al. (2023)
	Salvianolic acid B	Active the NRF2 signalling pathway, upregulate the expression of SLC7A11, GPX4, FPN and FTH1	SD rats	–	Shen et al. (2022a)
	Curcumin	Active the NRF2/HO-1 signalling pathway, upregulate the expression of GPX4	New Zealand rabbits	H9c2 cells	Wei et al. (2022)
		Upregulate the expression of GPX4, downregulate the expression of ACSL4	Wistar rats	–	Kar et al. (2023)
	Resveratrol	Upregulate the expression of FTH1 and GPX4, downregulate the expression of TFR1	SD rats	H9c2 cells	Li et al. (2022a)
		Upregulate the levels of GSH, upregulate the expression of GPX4 and SLC7A11, downregulate the levels of Fe^2 +^, MDA and ROS	SD rats	H9c2 cells	Liu et al. (2022)
Saponins	Ginsenoside Re	Upregulate the levels of GSH, upregulate the expression of SLC7A11, downregulate the expression of miR-144-3p	WKY rats	H9c2 cells	Ye et al. (2023)
	Astragaloside IV	Active the NRF2 signalling pathway, upregulate the expression of GPX4, downregulate the expression of NOX	SD rats	–	Luo et al. (2021)
	Ophiopogonin D	Upregulate the expression of GPX4 and FTH1, downregulate the expression of TFR1, COX2, NOX1 and ACSL4	–	H9c2 cells	Lin et al. (2021a)
	Saikosaponin A	Upregulate the levels of GSH, upregulate the expression of GPX4 and SOD, downregulate the levels of MDA, downregulate the expression of ACSL4	–	HUVECs	Huang et al. (2022)
	Aralosides and Araloside A	Upregulate the expression of NR3C1 and SLC7A11, downregulate the expression of p53	–	AC16 cells	Liang et al. (2022b)
Others	Tanshinone IIA	Active the NRF2/HO-1 signalling pathway, upregulate the expression of GSH-Px, downregulate the levels of MDA	–	H9c2 cells	Yang et al. (2020)
		Active the NRF2 signalling pathway, upregulate the levels of GSH and SLC7A11, downregulate the levels of ROS	–	HCAECs	He et al. (2021)
	Geniposide	Active the GRSF1/GPX4 signalling pathway, upregulate the expression of FTH1, downregulate the expression of TFR1	SD rats	Primary cardiomyocytes, H9c2 cells	Shen et al. (2022b)
	Thymoquinone	Active the NRF2/HO-1 signalling pathway, upregulate the expression of GPX4 and FTH1	C57BL/6j mice	–	Luo (2022)

### 6.1 Alkaloids

Alkaloids are a class of nitrogen-containing organic compounds, many of which have complex cyclic structures and are mostly alkaline or neutral (Li X. et al., 2022). Alkaloids have powerful anti-inflammatory, antibacterial, antioxidant, and antitumor effects (Liu et al., 2019), and play an important role in the treatment of CVDs. Matrine is an alkaloid isolated from TCMs such as *Sophora flavescens* Aiton and *Euchresta japonica* Hook. f. ex Regel, and it has been confirmed to alleviate oxidative stress and cell death in various CVDs (Sun et al., 2022). In a mouse model of sepsis-induced myocardial injury, matrine protects the damaged myocardium by activating the PI3K/AKT pathway to upregulate GPX4 expression and downregulate ACSL4 expression to inhibit ferroptosis (Xiao et al., 2023). Berberine is an isoquinoline alkaloid extracted from TCMs such as *Coptis chinensis* Franch. and *Phellodendron amurense* Rupr. (Song et al., 2023), and it has strong anti-inflammatory and antioxidant activities and cardiovascular protective effects (Cicero and Baggioni, 2016). Yang et al. (Yang K.-T. et al., 2022) found that in a cardiomyocyte ferroptosis model induced by erastin and RSL3, berberine could reduce the accumulation of ROS and lipid peroxidation. *In vivo* experiments also showed that berberine reduced the levels of MDA and iron in rats, downregulated the expression of TFR1 and p53, and upregulated the expression of NRF2, FTH1, and GPX4 to reduce cardiotoxicity (Song et al., 2023).

### 6.2 Polyphenols

Polyphenolic compounds are widely present in plants and have diverse structures, but all consist of a phenyl ring combined with one or more hydroxyl groups. According to their structures, polyphenols can be divided into flavonoids, phenolic acids, stilbenes, and lignans (Cheng et al., 2017; Singla et al., 2019; Lesjak et al., 2022). Polyphenols have anti-inflammatory and antioxidant capacities, contribute to cardiovascular health, and can neutralise free radicals by providing electrons or hydrogen atoms to reduce oxidative damage. Polyphenols are also metal chelators, which can chelate Fe^2+^, inhibit the Fenton reaction, and reduce the production of ROS (Cheng et al., 2017).

Flavonoid compounds typically possess a C6–C3–C6 backbone structure and are subdivided into flavones, flavonols, flavanones, flavanols, isoflavones, and anthocyanins, among others (Serafini et al., 2010; Khoddami et al., 2013). Baicalin, a flavonoid glycoside isolated from the roots and stems of the TCM *Scutellaria baicalensis* Georgi, inhibits TFR1 and NCOA4 expression, reducing ferroptosis and ACSL4 expression and thereby alleviating myocardial I/R injury (Fan et al., 2021). Furthermore, baicalin exerts antioxidant effects by enhancing GPX4 activity and thereby mitigating AS (Wu et al., 2018). Naringenin is a flavanone compound, and Xu et al. (Xu et al., 2021) found that in a rat myocardial I/R injury model, naringin increased the expression of NRF2, SLC7A11, GPX4, FTH1, and FPN, inhibited ferroptosis, and alleviated myocardial I/R injury. In hypoxia/reoxygenation-induced H9c2 cells, erastin reversed the protective effect of naringenin on the cells. Puerarin, an isoflavone extracted from the TCM Puerariae Lobatae Radix, has been widely used in the treatment of CVDs (Zhou et al., 2014). Puerarin increases FTH1 and GPX4 expression, reduces NOX4 expression, decreases lipid peroxidation, and inhibits myocardial cell loss in HF (Liu et al., 2018). Zhou et al. (Zhou et al., 2022) found that puerarin activated the AMPK signalling pathway, increased GPX4 and ferritin expression, reduced ACSL4 and TFR1 expression, inhibited ferroptosis, and protected against sepsis-induced myocardial injury. Moreover, puerarin was shown to inhibit ferroptosis and reduce myocardial I/R injury by decreasing ROS and MDA production and increasing GSH and GPX4 levels (Ding et al., 2023). Cyanidin-3-glucoside, a natural anthocyanin, possesses potent antioxidant activity due to two hydroxyl groups on its B-ring (Tan et al., 2019). Cyanidin-3-glucoside downregulates NCOA4 and TFR1 expression, upregulates FTH1 and GPX4 expression, inhibits ferroptosis, and thus alleviates myocardial I/R injury (Shan et al., 2021). Icariin, an isopentenyl flavonoid compound, is the main active ingredient of the TCM *Epimedium brevicornum* Maxim. (He et al., 2020). Liu et al. (Liu X.-J. et al., 2021) found that icariin activated the NRF2/HO-1 signalling pathway, reduced Fe^2+^ and ACSL4 levels, increased GPX4 levels, and alleviated myocardial cell ferroptosis. Yu et al. (Yu et al., 2023) found that icariin alleviated ethanol-induced atrial remodelling and reduced susceptibility to atrial fibrillation by activating the SIRT1/NRF2/HO-1 signalling pathway, increasing GPX4 and SLC7A11 expression, and inhibiting ACSL4 and p53 expression. Additionally, icariin delays AS by increasing GPX4 and FTH1 expression and reducing TFR1 expression (Wang et al., 2023).

Salvianolic acid B, a phenolic acid compound, is the main active ingredient of the TCM *Salvia miltiorrhiza* Bunge and has been widely used to treat cardiovascular and cerebrovascular diseases (Shi et al., 2019). Shen et al. (Shen et al., 2022a) established a MI model by ligating the left anterior descending coronary artery in rats and found that salvianolic acid B activated the NRF2 signalling pathway, increased SLC7A11, GPX4, FPN, and FTH1 expression, inhibited ferroptosis, and alleviated MI.

Curcumin, a diphenylheptane compound derived from the rhizome of the plant *Curcuma longa* L., has been confirmed to possess anti-inflammatory, antioxidant, hypoglycaemic, wound-healing, antibacterial, and antitumour activities (Lesjak et al., 2022). In a diabetic rabbit model, curcumin inhibits diabetes-induced myocardial cell ferroptosis by activating the NRF2/HO-1 signalling pathway and increasing GPX4 expression (Wei et al., 2022). Kar et al. (Kar et al., 2023) found that curcumin inhibited myocardial cell ferroptosis and alleviated I/R injury by reducing ACSL4 expression and increasing GPX4 expression.

Resveratrol, a member of the stilbene family, is a natural polyphenol that protects the cardiovascular system against vascular wall oxidation, inflammation, and thrombosis (Bonnefont-Rousselot, 2016; Lesjak et al., 2022). Both *in vivo* and *in vitro* experiments have shown that resveratrol inhibits ferroptosis and protects against myocardial I/R injury by reducing Fe^2+^ levels, downregulating TFR1 expression, and increasing FTH1 and GPX4 expression (Li T. et al., 2022). In a rat MI model, resveratrol alleviates MI-related myocardial injury and fibrosis by increasing GSH levels, upregulating GPX4 and SLC7A11 expression, and reducing Fe^2+^ and MDA levels and ROS accumulation (Liu et al., 2022).

### 6.3 Saponins

Saponins are a class of natural glycosides that are divided into triterpenoid and steroid saponins and are widely found in TCMs such as *Panax ginseng* C. A. Mey., *Astragalus membranaceus* var. *mongholicus* (Bunge) P. K. Hsiao, *B. chinensis* DC., and *Anemarrhena asphodeloides* Bunge. Saponins possess various biological activities, including anti-inflammatory, antiviral, immunomodulatory, cardiovascular protective, and anticancer effects (Xu et al., 2016). Ginsenoside Re, an active ingredient of the TCM *P. ginseng*, protects against myocardial I/R injury by inhibiting miR-144-3p expression, upregulating SLC7A11 expression, and increasing GSH levels to inhibit ferroptosis (J et al., 2023). Astragaloside IV activates the NRF2 signalling pathway, thereby increasing GPX4 expression, and reduces the expression of the positive regulator of ferroptosis NOX, thereby inhibiting Adriamycin-induced myocardial ferroptosis (Luo et al., 2021). Ophiopogonin D inhibits the expression of the ferroptosis-related proteins TFR1, cyclooxygenase 2 (COX2), NOX1, and ACSL4, increases GPX4 and FTH1 expression, and alleviates ferroptosis in rat myocardial cells (Lin Y. et al., 2021). Saikosaponin A, a triterpenoid saponin isolated from the TCM *Bupleurum chinensis*, increases GSH levels and SOD activity, reduces MDA levels, upregulates GPX4 expression, downregulates ACSL4 expression, and inhibits ferroptosis in a concentration-dependent manner, thus showing promise as a novel option for AS prevention and treatment (Huang et al., 2022). Aralosides and Araloside A inhibit hypoxia/reoxygenation-induced ferroptosis in AC16 cardiomyocytes by upregulating NR3C1 and SLC7A11 expression and downregulating p53 expression (Liang F. et al., 2022).

### 6.4 Others

Tanshinone IIA, a natural diterpene quinone compound, exhibits various biological activities, including anti-atherosclerosis effects, and alleviates angina and MI (Fang Z.-Y. et al., 2020). In a rat cardiomyocyte model of H_2_O_2_-induced oxidative damage, tanshinone IIA protects cardiomyocytes by activating the NRF2/HO-1 signalling pathway, enhancing GSH-Px activity, and reducing MDA activity (Yang et al., 2020). Tanshinone IIA also activates the NRF2 signalling pathway, increases GSH and SLC7A11 levels, reduces ROS production, protects the vascular endothelium, and delays AS development (He et al., 2021). Geniposide, a natural iridoid glycoside compound mainly derived from the TCM *Gardenia jasminoides* J. Ellis (Zhou et al., 2019), alleviates myocardial injury caused by MI by activating the GRSF1/GPX4 signalling pathway, increasing FTH1 expression, and reducing TFR1 expression (Shen et al., 2022b). Thymoquinone, a monoterpenoid compound (Kohandel et al., 2021; Talebi et al., 2021), activates the NRF2/HO-1 signalling pathway, increases GPX4 and FTH1 expression, and inhibits the cardiotoxic effects of doxorubicin (Luo, 2022).

## 7 Conclusion and outlook

Although CVDs are multifactorial and complex diseases involving multiple mechanisms, diabetes is a major risk factor. Increasing evidence suggests that ferroptosis plays an important role in diabetes and its cardiovascular complications (Yang and Yang, 2022), thus providing new therapeutic targets for diabetes-related cardiovascular complications.

In this review, we summarised the prerequisites for driving ferroptosis, including iron overload, ROS generation, and lipid peroxidation, as well as the defence mechanisms against ferroptosis, including the GPX4/GSH, FSP1/CoQ_10_H_2_, DHODH/CoQ_10_H_2_, and GCH1/BH4 systems. These genes could potentially serve as therapeutic targets for diabetic CVDs. Despite certain advancements in the study of ferroptosis mechanisms, challenges remain; in particular, specific biomarkers are currently lacking for ferroptosis, and many studies assess ferroptosis based on Fe^2+^ levels, ROS accumulation, and GPX4 expression. However, there are other forms of iron-dependent cell death that are distinct from ferroptosis, and ROS accumulation also occurs in oxidative stress. Therefore, finding specific biomarkers for ferroptosis is of great value. Furthermore, diabetic CVDs involve various mechanisms, including inflammation, oxidative stress, necrosis, apoptosis, pyroptosis, and ferroptosis. Identifying which mechanism dominates the development of disease and finding how to combine medications are problems that need to be solved in the future. Lastly, various organelles and proteins participate in the regulation of ferroptosis, increasing the difficulty of selecting targets for inhibiting ferroptosis. Different diseases have different therapeutic targets. Promoting ferroptosis in tumour cells helps inhibit and kill tumours, but normal cells, such as cardiomyocytes, pancreatic β-cells, and neurons, are also sensitive to ferroptosis. Determining how to selectively regulate ferroptosis is a major issue that requires extensive future research.

TCM has a long history of treating CVDs and has achieved a significant efficacy. The active ingredients of TCMs have various biological functions, and the discovery and continuous exploration of ferroptosis mechanisms provide a new theoretical basis for the treatment of CVDs with TCMs. Research findings on the treatment of CVDs through ferroptosis-related mechanisms by active ingredients of TCMs, such as alkaloids, polyphenols, and saponins, are summarised in Table 1. These compounds can act on various ferroptosis-related signalling pathways or regulatory proteins, protecting cardiomyocytes or vascular endothelial cells from ferroptosis. However, there are many problems to be solved in the treatment of CVDs by regulating ferroptosis with TCMs. First, the extraction and purification of active ingredients of TCMs are relatively complex processes that are affected by the environment, the quality of medicinal materials, and the process flow. Second, the treatment of diseases with TCMs does not depend on a specific active ingredient but often involves multiple ingredients working together, making it difficult to elucidate their mechanisms of action. Third, most of the current research on the mechanisms of active ingredients of TCMs intervening in ferroptosis is concentrated on animal- and cell-based experiments, while there is a lack of high-quality clinical research, especially randomised controlled trials. Fourth, MI has a sudden onset and a certain intervention time window, which raises a question whether TCMs targeting ferroptosis can exert their effects in time. Fifth, although the side effects of TCMs are small, chronic diseases such as chronic HF require long-term medication, and its safety still needs a large amount of clinical research for evaluation.

In conclusion, ferroptosis participates in the development of diabetes and CVDs, and a large amount of evidence shows that targeting ferroptosis in the treatment of CVDs by active ingredients of TCMs has certain advantages. In the future, it is necessary to continue research to improve the understanding of the signalling pathways and mechanisms related to ferroptosis, to provide new directions for the development of ferroptosis inhibitors, and thus provide new strategies for the treatment of diabetic CVDs.

## Figures and Tables

**Table:** 

^•^ **OH**	Hydroxyl radicals
**ACSL4**	Acyl-coenzyme A synthetase long chain family member 4
**AS**	Atherosclerosis
**ATF3**	Activating transcription factor 3
**BH4**	Tetrahydrobiopterin
**CoA**	Acyl-coenzyme A
**CVDs**	Cardiovascular diseases
**DCM**	Diabetic cardiomyopathy
**DHODH**	Dihydroorotate dehydrogenase
**DMT1**	Divalent metal transporter 1
**ERS**	Endoplasmic reticulum stress
**Fe^2+^**	Ferrous iron
**Fe^3+^**	Ferric iron
**FPN**	Ferroportin
**FSP1**	Ferroptosis suppressor protein 1
**FTH1**	Ferritin heavy chain 1
**FTL**	Ferritin light chain
**GCH1**	GTP cyclohydrolase 1
**GPX4**	Glutathione peroxidase 4
**GSH**	Glutathione
**GSSG**	Oxidized glutathione
**HF**	Heart failure
**HO-1**	Haem oxygenase 1
**HSPB1**	Heat shock protein family B (small) member 1
**I/R**	Ischemia/reperfusion
**IRE**	Iron-responsive element
**IRP**	Iron-regulatory protein
**LOXs**	Lipoxygenases
**LPCAT3**	Lysophosphatidylcholine acyltransferase 3
**MDA**	Malondialdehyde
**MI**	Myocardial infarction
**MUFAs**	Monounsaturated fatty acids
**NADPH**	Reduced nicotinamide adenine dinucleotide phosphate
**NCOA4**	Nuclear receptor coactivator 4
**NOX4**	NADPH oxidase 4
**NRF2**	Nuclear factor-erythroid 2-related factor 2
**PLs**	Phospholipids
**PUFA-PL-OHs**	PUFA phospholipid alcohols
**PUFA-PL-OOHs**	PUFA phospholipid hydroperoxides
**PUFA-PLs**	Phospholipids containing polyunsaturated fatty acids
**PUFAs**	Polyunsaturated fatty acids
**ROS**	Reactive oxygen species
**STEAP3**	Six-transmembrane epithelial antigen of prostate 3
**System x_c_^–^**	The cystine-glutamate antiporter
**T2DM**	Type 2 diabetes mellitus
**TF**	Transferrin
**TFR1**	Transferrin receptor protein 1
**TCM**	Traditional Chinese medicine
**UTRs**	Untranslated regions
